# Temperate climate malaria in nineteenth century Denmark

**DOI:** 10.1186/s12879-022-07422-2

**Published:** 2022-05-04

**Authors:** Mathias Mølbak Ingholt, Tzu Tung Chen, Franziska Hildebrandt, Rasmus Kristoffer Pedersen, Lone Simonsen

**Affiliations:** 1 PandemiX Center, Department of Science and Environment, Roskilde University, Universitetsvej 1, 4000 Roskilde, Denmark; 2 Regional Climate Group, Department of Earth Sciences, University of Gothenburg, 405 30 Gothenburg, Sweden; 3 Department of Molecular Biosciences, The Wenner-Gren Institute, Stockholm University, Stockholm, Sweden

## Abstract

**Background:**

*Plasmodium vivax* was endemic in northern Europe until the early twentieth century. Considering climate change and the recent emergence of other vector borne diseases in Europe, historical insight into the relationship between malaria and environmental factors in northern Europe is needed. This article describes malaria epidemiology in late-nineteenth century Denmark.

**Methods:**

We described the seasonality and spatial patterns of malaria, and the relationship of the disease with environmental factors such as soil types, clay content and elevation for the period 1862–1914. We studied demographic and seasonal patterns and malaria mortality in the high-morbidity period of 1862–1880. Finally, we studied the relationship between malaria seasonality and temperature and precipitation using a Spearman correlation test.

**Results:**

We found that the highest incidence occurred in eastern Denmark. Lolland-Falster medical region experienced the highest incidence (14.5 cases per 1000 pop.) and Bornholm medical region experienced the lowest incidence (0.57 cases per 1000 pop.). Areas with high malaria incidence also had high soil clay content, high agricultural production, and Lolland-Falster furthermore has a low elevation. Malaria incidence typically peaked in May and was associated with high temperatures in July and August of the previous year but not with precipitation. The case fatality rate was 0.17%, and the disease affected both sexes and all age groups except for infants. In 1873, a large epidemic occurred following flooding from a storm surge in November 1872.

**Conclusions:**

Malaria gradually declined in Denmark during our study period and had essentially disappeared by 1900. The high adult and low child morbidity in 1862–1880 indicates that malaria was not highly endemic in this period, as malaria is most frequent among children in highly endemic areas today. The association of high malaria incidence in spring with warmer temperatures in the previous summer suggests that transmission took place in the previous summers. The close geographical connection between malaria and soil types, agricultural production and elevation suggests that these factors are detrimental to sustain endemic malaria. Our findings of a close connection between malaria and environmental factors such as climate and geography provides insights to address potential reintroduction of malaria in temperate climates.

## Background

Although malaria caused by *Plasmodium vivax* is now mostly endemic to tropical climates, there was a substantial burden of *P. vivax* malaria in nineteenth century northern Europe [1–9]. In Denmark, malaria incidence declined in the second half of the nineteenth century, and by the first decades of the twentieth century, it was eliminated as an endemic disease [10]. Climate change raises the concern that malaria may again become endemic in countries outside the tropics, possibly including Scandinavia [11, 12]. In this article, we describe the epidemiology of malaria in late nineteenth century Denmark to improve our understanding of the influence of environmental factors on historical malaria transmission in northern Europe.

Research into the historical occurrence of malaria in northern Europe can be dated back to the late nineteenth century, where it was conducted by doctors [9, 13]. In recent times, it has however been the domain of historians and demographers. Whereas the historical epidemiology of malaria is well-described in England [14, 15], the Netherlands [9, 10], Finland [16, 17] and Sweden [2, 18], the evidence from Denmark is more anecdotal. The medical doctor Carl Adam Hansen described Danish malaria in 1886 [13], and in 1921, the entomologist Carl Wesenberg-Lund described the Danish malaria vectors [19]. Since then, Danish research has mostly focused on an exceptional lethal epidemic in 1831 suspected to be malaria [13, 18, 20–22].

### The malaria diagnosis

The diagnoses associated with malaria in late nineteenth century Denmark were *“Febris intermittens”* in Latin and *“koldfeber”* in Danish. In this period, doctors based the *koldfeber* diagnosis on patients exhibiting a combination of fever paroxysms of heat, chills and sweat, and spleen and liver enlargement. Complications included edema, jaundice, and gastric symptoms [23–26]. By the time the *Plasmodium* parasite was discovered in 1880 and its relationship with mosquitoes described in the 1890’s [27], malaria had nearly disappeared from Denmark [10].

*Koldfeber* symptoms were consistent with contemporary vivax malaria. This suggests that the historical *koldfeber* diagnosis almost certainly was malaria. In addition, quinine, an established contemporary anti-malarial, was effective against *koldfeber* [23–26]. By the early twentieth century, Danish doctors were able to isolate *Plasmodium* parasites in blood smears from suspected *koldfeber* patients, including from a woman who had never travelled outside Denmark [3–6].

### The northern European *P. vivax* and its vector

The northern European *P. vivax* has been associated with at least two mosquito vectors, both belonging to the *Anopheles maculipennis* complex. One was *An. atroparvus,* which in the nineteenth century existed in countries bordering the North Sea and in southern Sweden but has disappeared from many of these countries today [28, 29]. The other was *An. messeae,* which is still common as far north as Finland [16, 28]. In Denmark, female anophelines first emerged from their hibernation sites in May before expanding in population size until the late fall, when they once more went into hibernation [19].

Since *P. vivax* parasites cannot successfully develop below 16 °C, the Danish temperate climate was a major limitation for the survival of the parasite [12]. Thus, the parasite required an efficient overwintering strategy. A phenotypic trait of *P. vivax* is its ability to form a dormant stage in the human liver known as a hypnozoite. Reactivation of these hypnozoites allows the parasite to induce the symptomatic stage of the disease long after primary infection [30–33]. An incubation period of up to 9 months in the human liver before the primary onset of malaria has also been described [7, 28]. Reactivation or incubation would allow the parasite to overwinter in the human host.

The biology of hypnozoite formation and the molecular cues for their reactivation remain largely unknown [32]. Factors believed to be associated with the hypnozoite reactivation include mosquito bites [34–36], genetic factors of the parasite [37, 38], parasite burden and putative communication [32], as well as factors intrinsic to the host [39].

There are currently two main hypotheses for how malaria transmission took place in northern Europe. The first hypothesis assumes that transmission occurred in winter, when infected mosquitoes would awake temporarily from hibernation to take blood meals from humans, transmitting the parasites and causing primary onset of malaria in the spring [16, 40, 41]. Under this hypothesis, occasional autumn outbreaks of malaria were assumed to be caused by relapses from activated hypnozoites [41]. However, entomological studies from the Netherlands have demonstrated that during the winter sporozoites in infected mosquitoes degenerate and the overall proportion of mosquitoes declines [9]. In addition, a study from southern England found that mature female mosquitoes disappear during the winter and do not return until March [42]. Taken together, these studies suggest that the hypothesis of winter transmission is unlikely. The second and widely accepted hypothesis assumes that transmission occurred during the summer, when mosquito populations were active and the temperatures warm. Transmission was followed either by immediate onset of malaria in the autumn, or by long-term dormancy until disease onset in the following spring [9, 12, 29, 41, 43, 44].

## Methods

### Malaria health data

We digitized data on malaria morbidity, stratified by year, calendar month, and regional geography from “Medicinalberetning for Kongeriget Danmark” [45] for the period 1862 to 1914. These reports contained malaria morbidity statistics from 1862, for which reason this year is chosen as the starting year. For data consistency, 1914 was chosen as end year of the study period, as the Danish medical regions were reorganized in 1915. Malaria was a reportable disease for the entire period. Data on age were recorded from 1868. Beginning in 1871, totals were also recorded by type of location, including province towns, rural districts and Copenhagen. For Fig. 5A showing parish level malaria incidence per 1000 population for Lolland-Falster, we included incidence data from a thesis by aforementioned Carl Adam Hansen [13].

### Demographic data

We extracted population data from the 1860, 1870, 1880, 1890, 1901, 1906 and 1911 censuses conducted by Statistics Denmark. In the censuses before 1901, population by medical region was not included; however, because medical region borders largely follow the Danish county borders, we reconstructed the medical regions’ populations from county population sizes. We estimated medical region population for inter-census years by linear interpolation.

### Malaria mortality data

Cause-specific infectious disease mortality data were available for Copenhagen and Danish province towns only. We extracted malaria mortality data for these places for the period 1871–1880 from an annual publication series by Statistics Denmark [46].

### Agriculture data

We obtained data on the proportion of land used for agricultural purposes (i.e., tilled land and grazing pastures) in Danish counties in 1876 from a survey by Statistics Denmark [47]. We estimated the percentage of land used for agriculture in each county and mapped it using the geographical information system software QGIS.

### Analysis

We mapped average geographical prevalence per 1000 population of malaria across Danish medical regions for 1863–1872 using the QGIS software and compared this visually with maps of agricultural intensity and soil types. We did not include 1862 because a large epidemic that year may have influenced the incidence pattern. We studied the relationship between malaria seasonality and the previous year’s monthly temperature and precipitation in the region of Lolland-Falster using Spearman correlation tests in the programming language R version 4.1.2. Next, we used the high-morbidity period 1862–1880 to further explore age patterns, the case fatality rate and seasonality.

## Results

### Temporal patterns

**Fig. 1:**
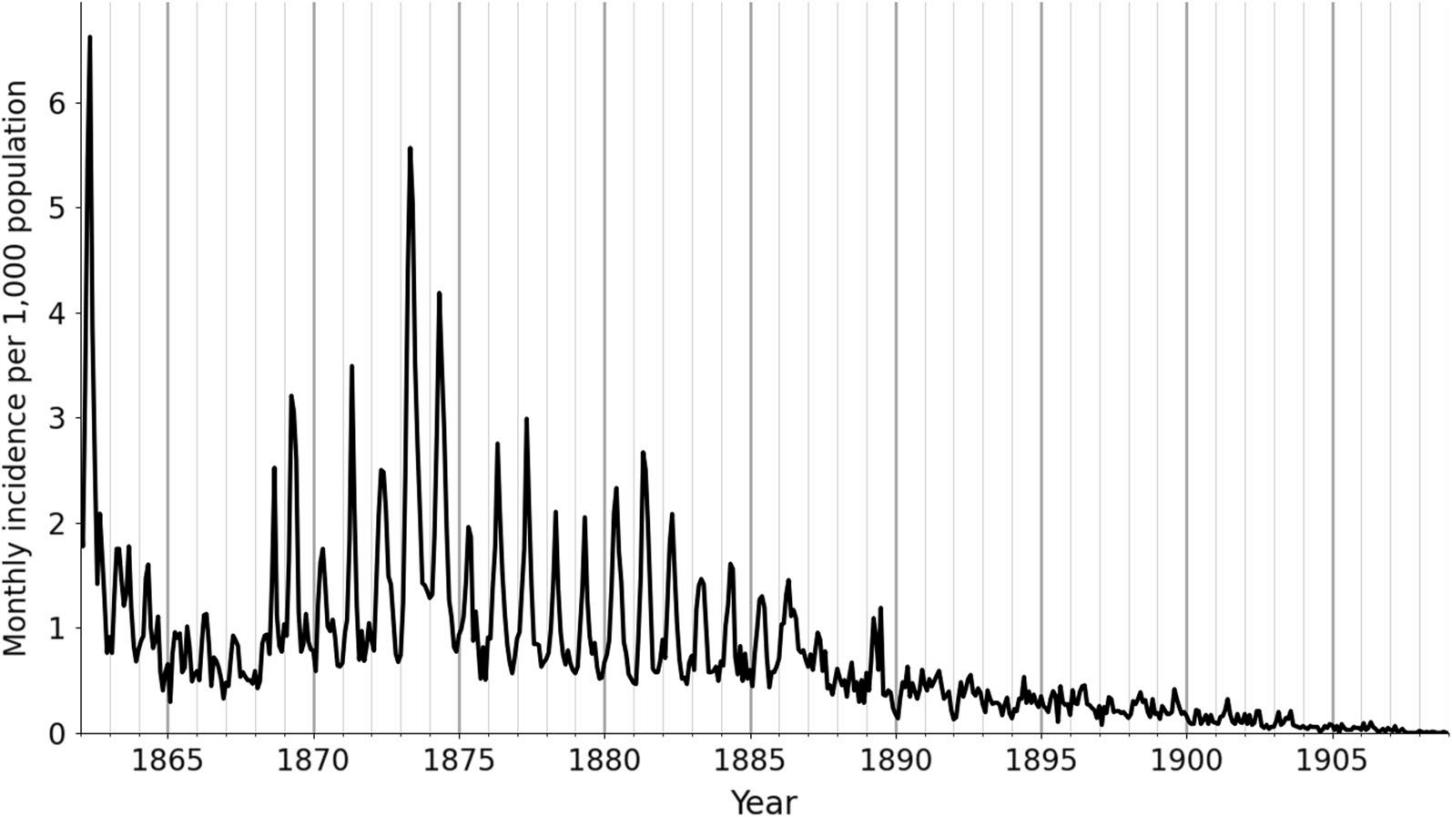
Monthly malaria incidence for Denmark during 1862–1908. National monthly malaria incidence per 1000 population for the period 1862–1908 in Denmark. Incidence was highest in the spring months and usually peaked in May. Severe malaria epidemics occurred in 1862 and 1873–1874

A total of 171,100 cases of malaria were recorded by physicians between 1862 and 1914. The annual number of cases declined more than a thousand-fold in these five decades, from a maximum of 38,096 in 1862, when Demark experienced a nationwide malaria epidemic [13], to a minimum of 33 in 1914 (Fig. 1).

**Fig. 2:**
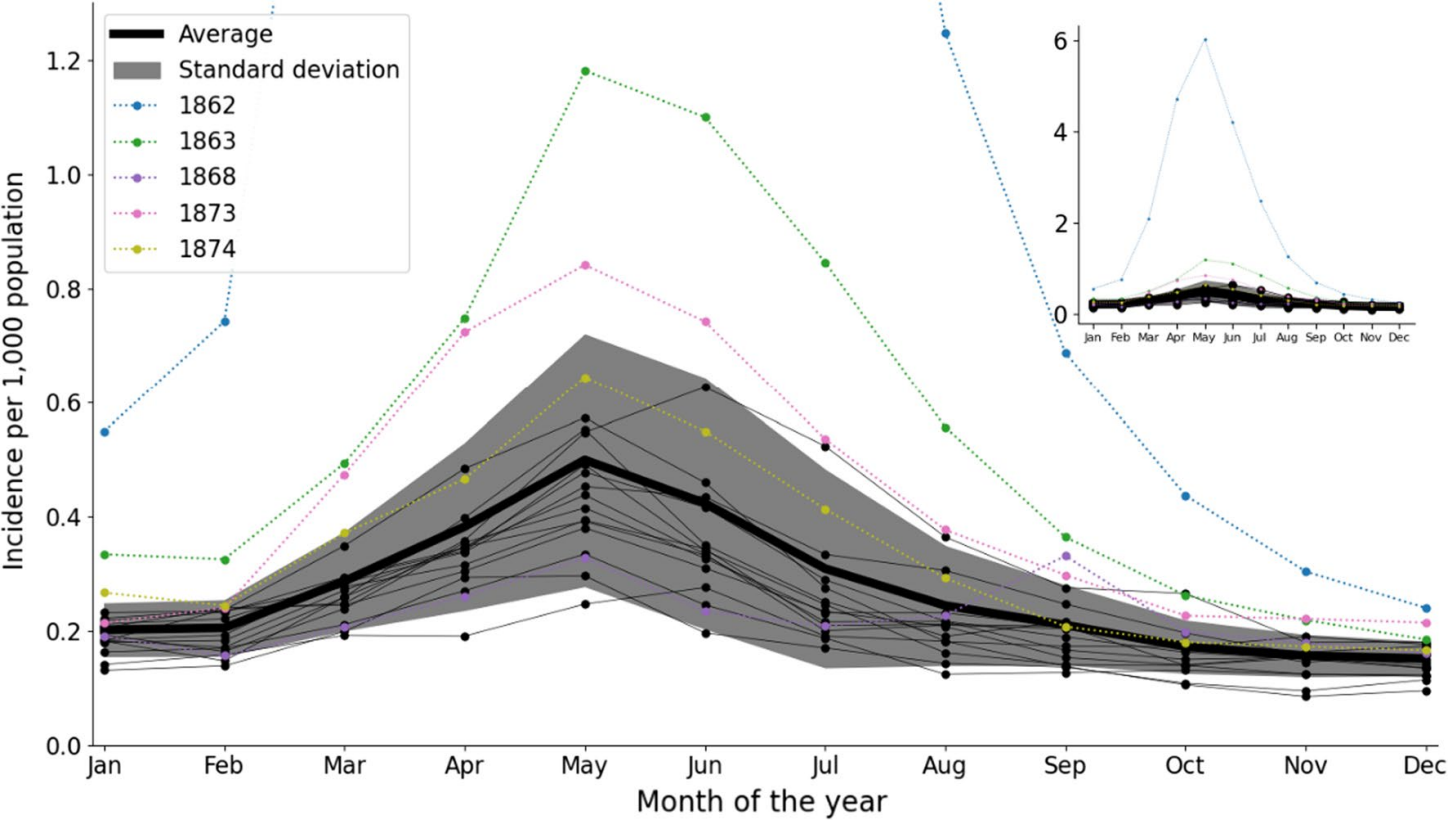
Seasonality of malaria in Denmark. Monthly malaria incidence per 1000 population in Denmark for each of the 19 years during 1862–1880. The seasonal incidence pattern of 1862 is included in the small figure in the upper right corner. The four years with highest annual incidence (1862, 1863, 1873 and 1874) and a rare year with a prominent September peak (1868) are highlighted in color. Black lines represent non-epidemic years. 1862 was excluded from the average (heavy black line)

Malaria exhibited a distinct seasonal pattern with incidence peaks in May (Figs. 1, 2). In some years, we observed minor increases in incidence in September. Most pronounced of these was 1868, where a September peak coincided with a particularly warm summer (Fig. 2).

**Fig. 3:**
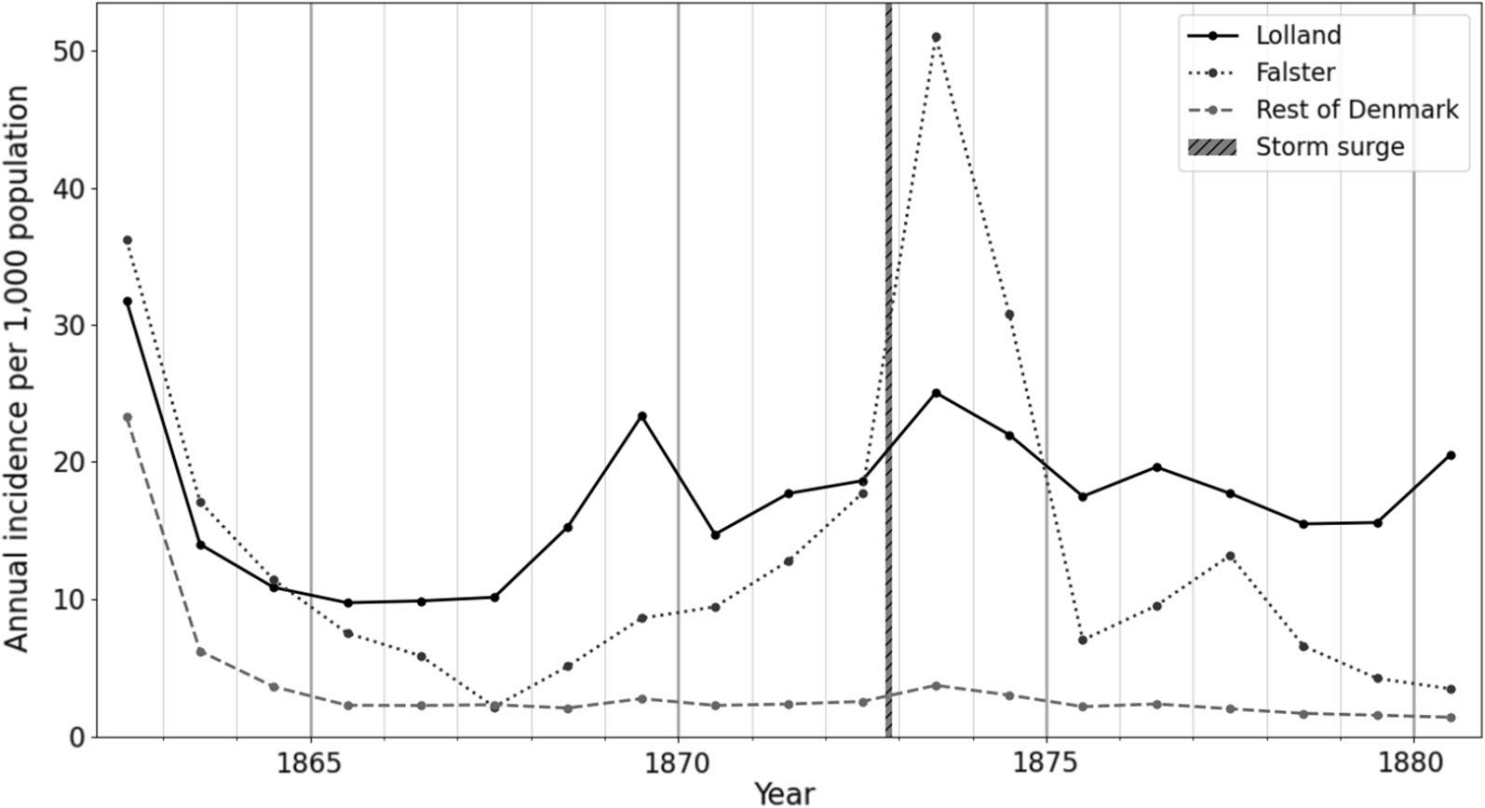
Association between the 1873 epidemic and the 1872 storm surge. Annual malaria incidence per 1000 population for medical districts in Lolland and Falster (part of Lolland-Falster medical region) and the rest of Denmark for 1862–1880. The highest incidence of the 1873 outbreak was observed in Falster, which had the most severe flooding

A storm surge in November 1872 flooded low-lying areas and islands throughout southeastern Denmark, including parts of Lolland-Falster and Funen. The most severe flooding occurred in Falster, where the island’s southern tip was temporarily separated from the rest of the island. Lolland experienced less extreme flooding [50]. In Falster, malaria incidence was three-fold higher in the spring of 1873 than in 1872, and a minor increase was observed in Lolland. No similar increase occurred in the rest of Denmark, where malaria was also endemic but no flooding had occurred (Fig. 3).

The Spearman correlation test of malaria incidence and temperatures showed a strong correlation between both annual and May malaria incidence and the previous year’s July and August temperatures (Table 1). The analysis also showed a significant negative statistical correlation between the winter temperatures and annual malaria incidence. No significant correlation was found with precipitation data (Table 1).

**Table 1:** Spearman correlation coefficients for association of malaria and temperatures and precipitation in Lolland-Falster

Time period in previous year	Spearman coefficient			
	Annual incidence and temperature	May incidence and temperature	Annual incidence and precipitation	May incidence and precipitation
Month				
January	− 0.24	− 0.09	0.34	− 0.25
February	− 0.22	− 0.24	− 0.22	− 0.25
March	0.44	0.40	0.14	0.16
April	− 0.11	0.00	− 0.02	− 0.11
May	0.12	0.12	− 0.10	− 0.02
June	0.26	0.38	0.08	− 0.03
July	0.58*	0.66*	0.11	0.02
August	0.50*	0.57*	− 0.34	− 0.23
September	0.17	− 0.33	0.23	0.04
October	− 0.04	− 0.03	0.23	0.23
November	− 0.14	0.02	− 0.09	− 0.04
December	− 0.01	0.05	0.16	0.09
Season				
Spring (M,A,M)	0.19	0.20	− 0.01	0.01
Summer (J,J,A)	0.53*	0.69*	− 0.10	− 0.19
Autumn (S,O,N)	− 0.16	− 0.10	0.23	0.17
Winter (D,J,F)	− 0.45*	− 0.41	− 0.17	− 0.18

*p ≤ 0.05

Spearman correlation coefficient between the preceding year’s monthly temperature and annual and May malaria incidence (per 1000 population, 27,210 cases in total) in Lolland-Falster during 1862–1880. MAM = March, April, May. JJA = June, July, August. SON = September, October, November. DJF = December, January, February

### Spatial patterns

**Fig. 4:**
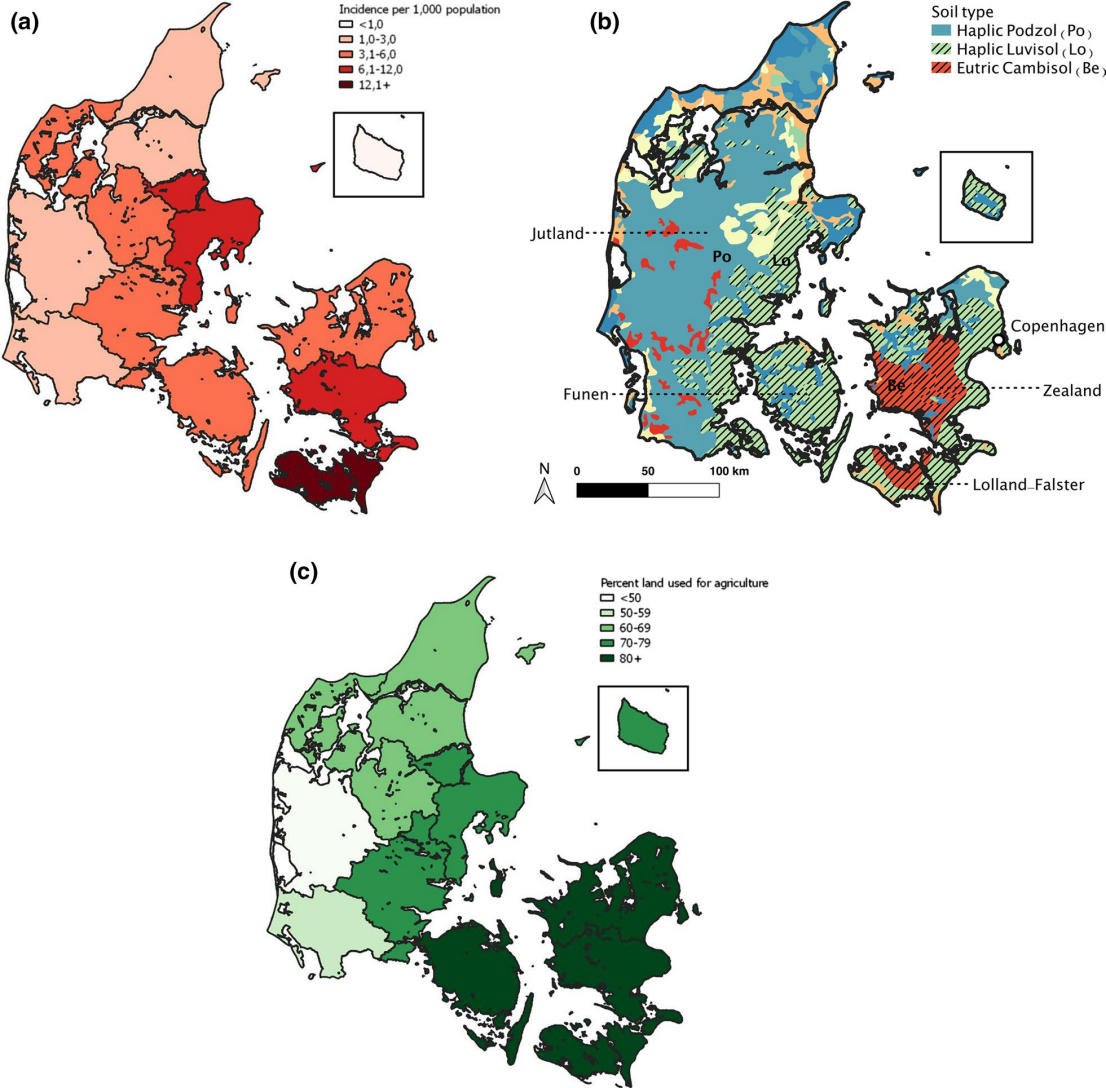
Maps showing (**A**) malaria incidence during 1863–1872 by medical region; (**B**) Soil type [65]; and (**C**) percentage total landmass used for cultivation or pasture, by county

**Fig. 5:**
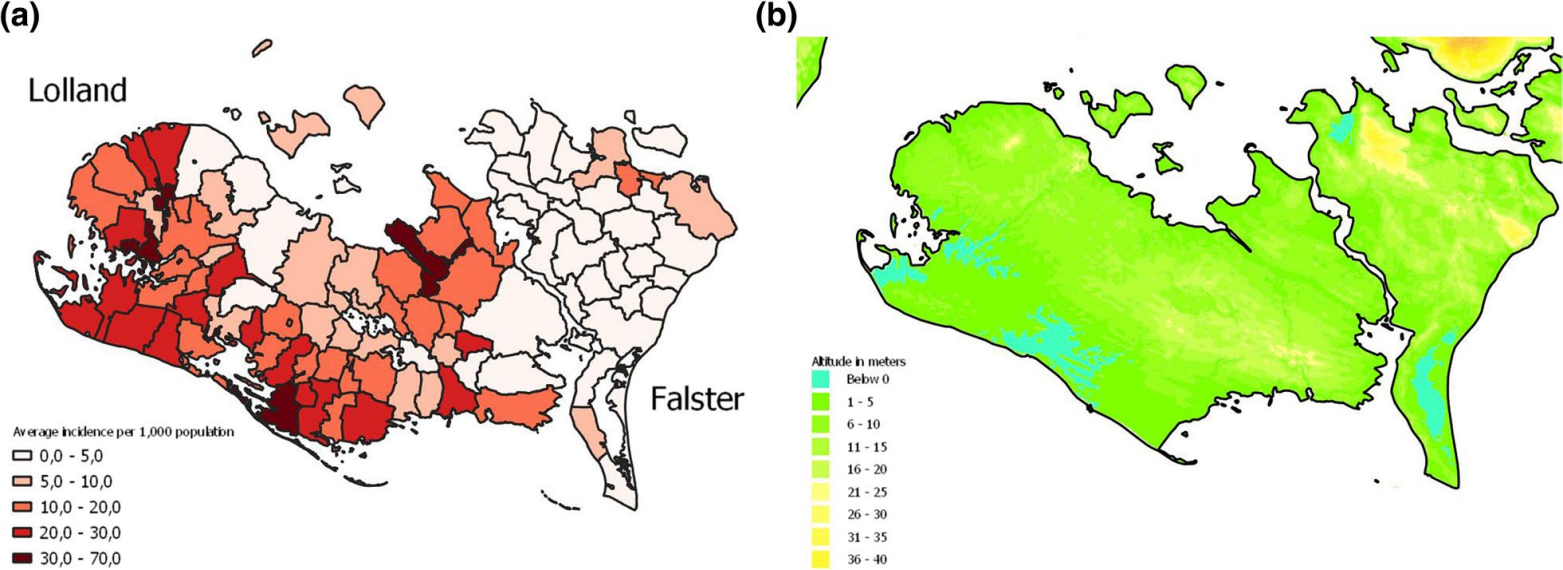
Maps showing (**A**) average annual malaria incidence per 1000 population by parish for Lolland-Falster during 1879–1883 and (**B**) elevation in meters above sea level [66]. Higher incidence occurred in low-lying wetlands and coastal areas of Lolland

During the period 1863–1872, the annual malaria incidence was highest in southeastern Denmark, particularly in Lolland-Falster (14.5 cases per 1000 population) and lowest in western Jutland and on the island of Bornholm (0.5 cases per 1000 population) (Fig. 4A). Regions with higher malaria incidence along the east coast of Jutland, Zealand and Lolland-Falster are largely covered by Luvisol and Cambisol soils with high clay content (Fig. 4B shaded areas, 84) and had a high agricultural production (Fig. 4C). Other parts of Denmark with low malaria incidence have predominantly Podzol soils with high sand content and simultaneously saw lower agricultural production (Fig. 4C). Within Lolland-Falster, malaria was most frequent in the parts of the island with the lowest elevation (Fig. 5A, B).

**Fig. 6:**
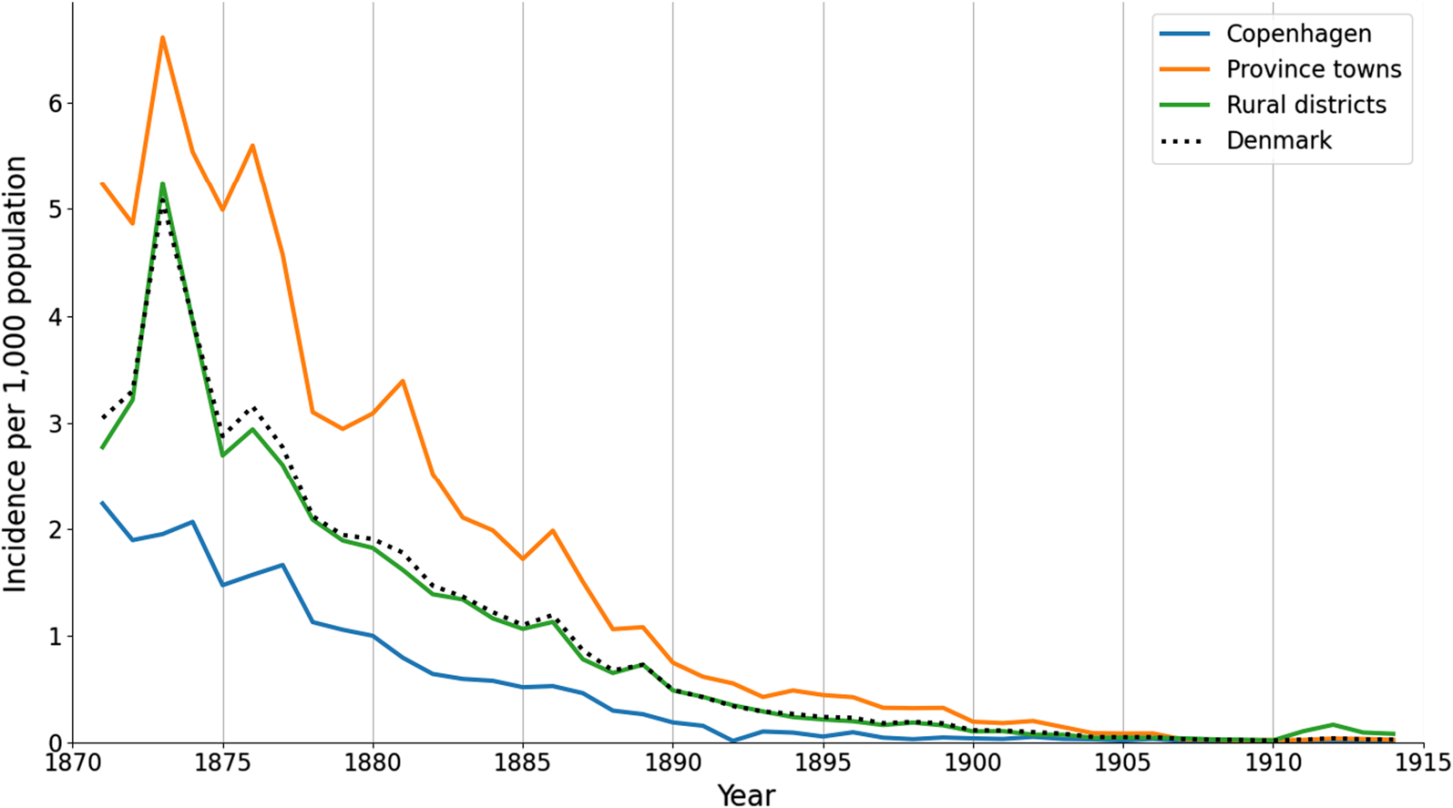
Annual malaria incidence by urban and rural areas. Annual malaria incidence per 1000 population for the period 1871–1914 in Copenhagen, the province towns, the rural districts and Denmark. Incidence was highest in the province towns, but the national incidence trend closely followed that of the rural districts

The reported malaria incidence was highest in the province towns and lowest in Copenhagen, while the large population in the rural areas had intermediate incidence rates (Fig. 6).

### Age, sex and mortality

**Fig. 7:**
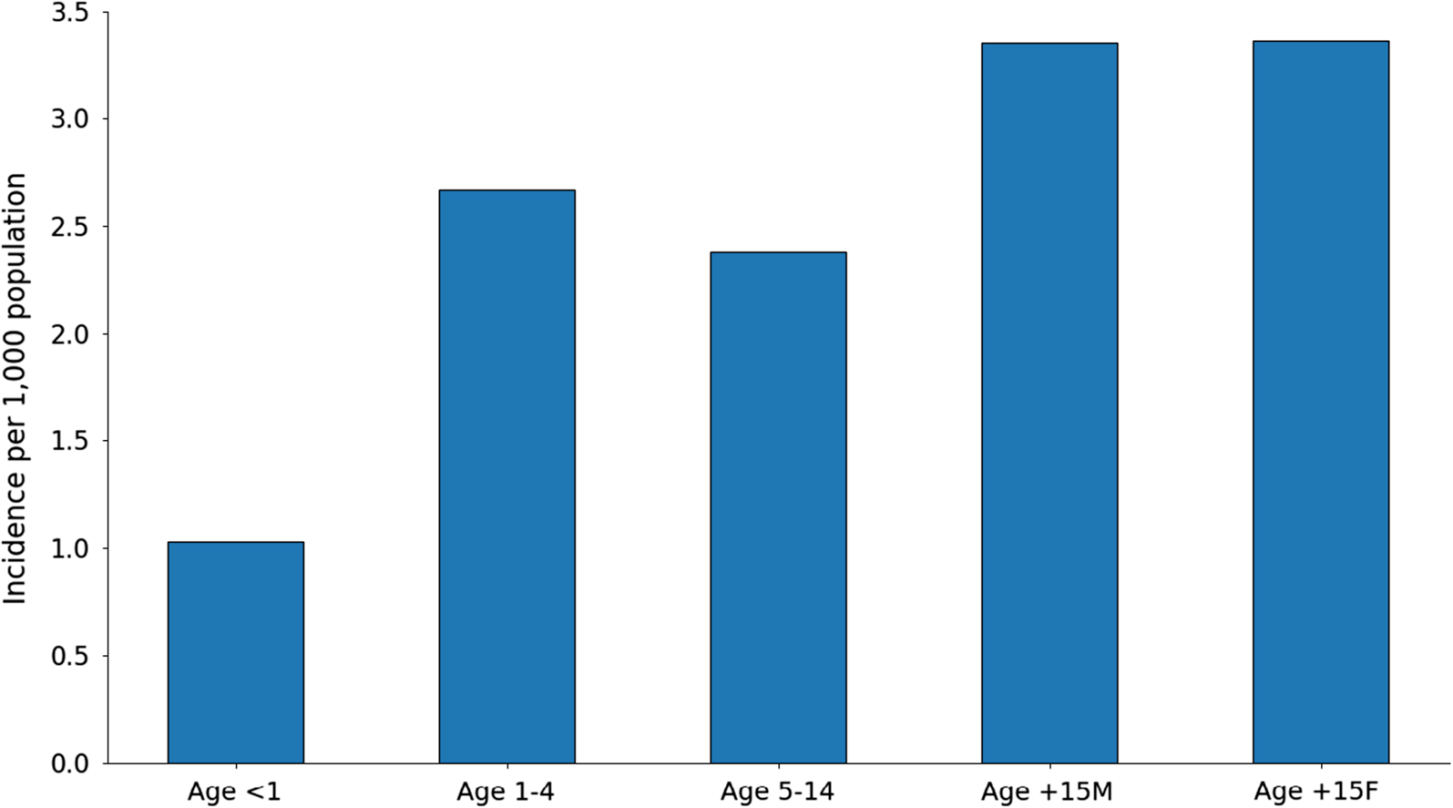
Malaria incidence by age and sex. Average malaria incidence per 1000 population for all of Denmark, by age groups (years) and sex (adults only; M = male, F = female), for the period 1868–1880

A total of 72,549 malaria cases were recorded during 1868–1880, with the highest incidence among adults of both sexes and lowest incidence among infants (Fig. 7). Between 1871 and 1880 in Copenhagen and the province towns, 15,064 malaria cases were reported but only 25 deaths were attributed to malaria, which corresponds to a case fatality rate (CFR) of 0.17%.

## Discussion

### Temporal patterns

Malaria incidence in Denmark declined in the late nineteenth century (Fig. 1) and was by 1900 no longer a public health threat. The spring peaks in malaria incidence are consistent with findings from studies of malaria mortality in other northern European countries in the eighteenth and nineteenth centuries, indicating that malaria was a spring disease in all of northern Europe [9, 16]. To sustain endemic transmission, the *Plasmodium* would overwinter as dormant hypnozoites in human livers and synchronize their reactivation with the emergence of female anophelines around May. This provides a potential explanation for these springtime epidemic peaks [28, 29, 44].

In northern Europe, the size of malaria epidemics depended on the previous year’s summer temperatures. In Denmark, springtime malaria incidence was positively correlated with higher temperatures during the previous July–August (Table 1), while in Finland and Sweden, malaria peak incidence was more closely correlated with June-July temperatures [2, 16]. Denmark’s lower latitude and warmer climate may explain the difference. Sweden and Finland have colder continental climates while Denmark has a warmer maritime climate [48, 49]. This means that the first mosquito generation emerge earlier in Denmark and have shorter larval periods [28]. Moreover, the second mosquito generation also hatched earlier, and would have already been able to transmit malaria by the end of August. We are unsure of the significance of the weaker negative correlation between annual malaria incidence and average temperature in the previous winter.

### Spatial patterns

The presence of clay-rich soil played a key role in the geographical malaria patterns of Denmark. Areas with clay soil were often used for intensive agriculture and *An. maculipennis* mosquitoes were especially abundant in agricultural areas such as pastures, stables and croplands [19, 51]. Pools of stagnant water form more easily and persist longer in soils with a high content of clay [52] and would have provided more and larger breeding sites for *Anopheles* mosquitos. Finally, higher moisture conditions in clay soils [54] can lead to a significant increase in biting rates of some *Anopheles* species [55]. The data in Fig. 4C is from 1876. In this period, Danish agriculture was intensifying with more dairy production [19]. A similar map with data from e.g., 1862 might have revealed less agricultural production in the Danish counties. The geographical pattern with the highest agricultural production in eastern Denmark would nevertheless have been similar to 1876 [13].

Elevation also impacted malaria incidence. Low-lying areas such as those in Lolland-Falster (Fig. 4A) were rich in coastal wetlands with high salt concentrations in the water and thus provided many optimal breeding sites for the *An. atroparvus* vector [44, 52, 53]. The geographical pattern of high malaria incidence in coastal wetlands has also been observed in south-east England and other parts of northern Europe [2, 9, 15, 17, 53].

Extreme weather conditions can increase epidemic severity. The summer of 1868 was as warm as the summer of 1872, yet only a minor malaria epidemic occurred in 1869 while the epidemic in 1873 was severe. We attribute the difference to flooding related to the November 1872 storm surge, a proposition also supported by the clear geographical association between the severity of flooding and the intensity of the malaria epidemic in southern Falster [50] (Fig. 3). The 1873 epidemic began in late winter, which was before mosquitoes came out of hibernation, and peaked in May/June. Furthermore, the 1872 storm surge took place after the 1872 mosquitoes had begun hibernation [19]. Therefore, neither the 1872 mosquitoes nor the first generation of mosquitoes in 1873 could have played crucial roles in the onset of the 1873 epidemic.

The first spring generation of female anophelines is small, and following generations expand over the summer. Excess salt water from the storm surge flooding and fresh water from rain could have mixed and created the brackish water necessary for *An. atroparvus* to breed. This could have expanded anopheline populations in the summer of 1873 and might explain the following epidemic in the spring of 1874. The parts of Sweden that experienced flooding during the storm surge [50, 56] also experienced malaria epidemics in 1873 and 1874 [2, 50]. Therefore, while it is clear that the 1873 and 1874 malaria epidemics in Denmark and Sweden were connected to the storm surge, the exact mechanisms that started the 1873 epidemic remains unclear.

### Urban versus rural settings

We found a lower malaria incidence in Copenhagen, by far the largest city in Denmark in the study period, than in rural areas, which is consistent with the contemporary pattern of *P. vivax* malaria being most prevalent in rural settings [57]. Industrial pollution has been shown to cause higher mortality and impaired development of *Anopheles* mosquitoes [28]. Copenhagen, however, was in the nineteenth century highly polluted with human and animal waste and other organic pollutants, which do not affect mosquitos the same way. Indeed, some species of *Anopheles* mosquitoes with the capacity to transmit malaria, including *An. plumberus,* lay eggs in organic waste [58], but the role of *An. plumberus* as historical malaria vector in Denmark is unclear.

Environmental conditions outside the Copenhagen’s urban core could explain some of the city’s malaria incidence. Copenhagen in the nineteenth century was surrounded by defensive freshwater moats, which may have served as reservoirs for *An. atroparvus* and *An. messeae.* As Copenhagen started to expand in the mid-nineteenth century, new neighborhoods were created that lay close to these moats, as well as surrounding bogs and creeks. Incidence during a malaria epidemic in 1856 was highest in these new neighborhoods, which suggests that the environmental conditions in these semi-rural neighborhoods were good for the known malaria vectors [59].

Malaria incidence was highest in Danish province towns (Fig. 6). Most such towns had a high population density but less than 10,000 inhabitants and were closely surrounded by fields and pastures for urban livestock grazing [60]. The combination of densely populated urban housing near livestock and rural environments with a high density of malaria vectors may explain the high malaria incidence. The higher incidence in the province towns could also be explained by a reporting bias. Doctors often practiced in the province towns and had less contact with the rural population. The medical system was private, leaving a large part of the rural population without access to trained medical doctors [61]. Rural malaria cases may therefore have been underreported.

### Malaria mortality and age

The relatively low CFR of 0.17% found in this study is in agreement with previous observations from England, the Netherlands and Germany that *P. vivax* malaria was not a highly lethal disease in the nineteenth century [10, 14]. A contemporary study of *P. vivax* mortality in Indonesia also found a low CFR (0.12 deaths per 1000 population) and that *P. vivax* infection is seldom the direct cause of death [62]. The relatively low lethality of malaria in Denmark suggests that a large mortality spike that occurred in Denmark in 1831 was not caused by malaria [13, 18, 21, 22]. Interestingly, malaria was a more lethal disease in Sweden and Finland in the 18th and early nineteenth centuries with a CFR of 0.85–3% in Finland but became less deadly during the nineteenth century [2, 17].

In contemporary settings with high incidence and constant transmission, malaria is most frequently reported among children [63]. However, the higher malaria incidence among adults in our study suggests that malaria was lower in incidence in late nineteenth century Denmark than in the high malaria regions today. In addition, the low infant and child incidence rates might be the result of underreporting due to contemporary physicians being unable to distinguish malaria from other childhood febrile diseases [24, 64], by less exposure, or by a combination of both reasons. The equal sex ratios in morbidity furthermore contrast with qualitative observations of male farm workers sleeping in stables and cowsheds being more exposed to mosquito bites [19].

## Conclusion

We studied the epidemiology of *P. vivax* malaria in late-nineteenth century Denmark based on well preserved longitudinal morbidity and mortality data. Malaria was most prevalent in the regions of Demark with high clay soil concentration and high agricultural production, particularly in the low-lying parts of Lolland-Falster. The reported incidence was highest in the province towns and rural settings, and lowest in Copenhagen. Malaria was a low-mortality disease with seasonal peaks in the spring, and typically in May.

The size of the spring peak depended on the previous summer temperatures, suggesting that summer was the peak period for malaria transmission. It is well understood that the hibernating behavior of mosquitoes during late fall and cold winters represented a major challenge for *Plasmodium* transmission in temperate climate countries. Presumably, the parasite overcame these challenges by forming dormant hypnozoites or otherwise incubating in human hosts over the winter. Hence, infected human hosts likely carried the *P. vivax* parasite into the next transmission season. We furthermore found that extreme weather events such as storm surges could also cause local malaria epidemics.

This historical epidemiological study identifies potential environmental factors that could have been associated with high malaria transmission in a temperate climate. It furthermore provides a complementary perspective to contemporary studies of malaria and may shed light on future challenges associated with the accelerating climate change impacts on reemergence of vector-borne diseases in Europe. Indeed, a historical insight is critical for scientists who are addressing or modelling scenarios for return of malaria in temperate climates as a consequence of climate change and severe weather incidents.

## Data Availability

The data used in this article comes from annual medical reports by the Danish Health Board. They are found in physical book format at the Royal Library in Copenhagen. This data is available from the corresponding author on reasonable request. Population census data for nineteenth century Denmark has been scanned by Statistics Denmark, and is available at: https://www.dst.dk/da/Statistik/Publikationer/VisPub?cid=19614. Malaria mortality data for Copenhagen and the province towns has also been scanned by Statistics Denmark, and can be found at: https://www.dst.dk/da/Statistik/Publikationer/VisPub?cid=19682. The parish-level malaria data used in Fig. 5A can be found at the Royal Library in Copenhagen.
